# Exergy Analysis of a Direct Contact Membrane Distillation (DCMD) System Based on Computational Fluid Dynamics (CFD)

**DOI:** 10.3390/membranes11070525

**Published:** 2021-07-13

**Authors:** Jihyeok Choi, Yongjun Choi, Juyoung Lee, Yusik Kim, Sangho Lee

**Affiliations:** School of Civil and Environmental Engineering, Kookmin University, 77, Jeongneung-ro, Seongbuk-gu, Seoul 027072, Korea; cjh6563@gmail.com (J.C.); choiyj1041@gmail.com (Y.C.); jyl20101530@gmail.com (J.L.); yskim920930@naver.com (Y.K.)

**Keywords:** direct contact membrane distillation (DCMD), temperature polarization, heat/mass transfer, computational fluid dynamics (CFD), exergy analysis

## Abstract

Understanding the energy efficiency of direct contact membrane distillation (DCMD) is important for the widespread application and practical implementation of the process. This study analyzed the available energy, known as exergy, in a DCMD system using computational fluid dynamics (CFD). A CFD model was developed to investigate the hydrodynamic and thermal conditions in a DCMD module. After the CFD model was verified, it was used to calculate the temperature polarization coefficient (TPC) and exergy destruction magnitudes under various operating conditions. The results revealed that slight decreases and increases in the TPC occurred with distance from the inlet in the module. The TPC was found to increase as the feed temperature was reduced and the feed and permeate flow rates were increased. The exergy destruction phenomenon was more significant under higher feed temperatures and higher flux conditions. Although the most significant exergy destruction in the permeate occurred near the feed inlet, the effect became less influential closer to the feed outlet. An analysis of exergy flows revealed that the efficiency loss in the permeate side corresponded to 32.9–45.3% of total exergy destruction.

## 1. Introduction

Membrane distillation (MD) is an emerging technique that is proposed as a promising alternative to multistage flash (MSF), multi-effect distillation (MED), and reverse osmosis (RO) processes [1,2,3]. In MD, a hydrophobic membrane is used as a barrier between the vapor and the liquid-phase water, which enables the production of high-quality fresh water from high-salinity water [3,4,5]. There are many advantages to using MD systems, including high rejection of non-volatile impurities, relatively low operating temperatures compared with other distillation technologies, and low hydraulic pressure requirements [2,6,7]. As the operation of MD systems is not constrained by the osmotic pressure of the feed water, the treatment of RO brine is possible [1,2,5].

There are four classifications of MD systems: direct contact membrane distillation (DCMD), air gap MD, vacuum membrane distillation (VMD), and sweeping-gas MD [8,9,10]. Owing to the simplicity and low cost of the DCMD technique, it has been investigated extensively [11,12]. However, one of the biggest problems with DCMD is its low energy efficiency associated with the temperature polarization (TP) phenomenon. Moreover, in the DCMD process, recovering the latent heat in the product water is more difficult than in the other MD configurations, which leads to a further reduction in the energy efficiency of DCMD systems.

Within the TP phenomenon, differences occur between the bulk temperature and the temperature of a membrane’s surface. Once TP occurs, the effective temperature difference across the membrane is reduced, leading to a decrease in driving force. TP is a major barrier to the widespread application of DCMD technology in industry [13,14,15]. Accordingly, much research has been conducted to elucidate and mitigate the TP phenomenon in MD systems [16,17,18,19], and the phenomenon has been analyzed and predicted in such systems using model equations under various operating conditions [20,21,22,23,24]. Additionally, heat transfer analysis was performed according to the angles for the membrane unit, and spacers and modules have been developed to increase the transfer of heat and mass, effectively reducing the TP phenomenon [25,26,27,28,29,30,31]. Although experimental methods have been applied to elucidate the TP phenomenon, computational fluid dynamics (CFD) have been increasingly used due to the capability of investigating the underlying physics and chemistry [32,33]. Eulerian methods have been widely adopted for CFD simulations, but Lagrangian methods have been also used as alternative approaches [34,35,36,37].

An analysis of the effectiveness of energy use in DCMD systems should consider the available energy instead of the total energy [38]. Based on the principles of both the first and second laws of thermodynamics, the exergy method of analysis can provide an accurate indicator of energy efficiency in DCMD systems [39]. Exergy is defined as the maximum amount of useful work that can be obtained as a system is brought to equilibrium with its environment [40]. Unlike energy, exergy is not conserved, owing to an increase in entropy [40]. The concept of exergy has been the focus of considerable attention from both academia and industry, and it has been used in a wide variety of thermal and chemical systems [40,41]. However, only limited research has been conducted to date on analyzing the exergy of DCMD systems, most of which has focused on solar-powered MD schemes [42] or VMD systems with vapor recompression [43].

This study proposes a novel approach to analyzing exergy in a DCMD system based on computational fluid dynamics (CFD). A CFD model was developed to calculate the velocity profiles, heat flows, and temperature distributions inside a laboratory-scale DCMD module. Based on the obtained results, the TP coefficient (TPC) and the magnitudes of exergy destruction within the system were evaluated under different operating conditions. To the best of the authors’ knowledge, this study reports the first application of CFD for analyzing the exergy flows in a DCMD system.

## 2. Theory

**Figure 1:**
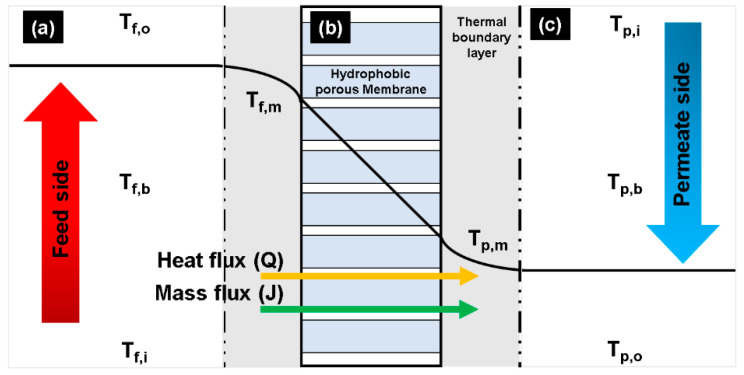
Schematic of heat/mass transfer through a hydrophobic porous membrane used in a DCMD process: (**a**) evaporation in the feed side, (**b**) transportation of water vapor across the membrane pore, and (**c**) condensation of water vapor in the permeate side.

Figure 1 presents a schematic representation of the heat and mass transfers in a DCMD membrane. A CFD model was developed to analyze this based on the following assumptions:

- Both the feed and permeate have a laminar flow regime and are in a steady state.
- Heat loss to the ambient environment is negligible.
- No chemical reaction occurs.
- Convective transport of water vapor via the membrane pore is negligible.

### 2.1. Computational Fluid Dynamics Model Equation

It is assumed that fluid flow through the two sides is both non-isothermal laminar and incompressible. The Navier–Stokes equations for the laminar flow in both channels can be expressed as follows [44]:

(1) $$\rho \left(u\cdot \nabla \right)u=\nabla \cdot \left[-pI+\mu \left(\nabla u+{\left(\nabla u\right)}^{T}\right)\right]+F,$$

(2) ρ∇·u=0,

(3) $$\{\begin{array}{c} \rho_{f}=\frac{100}{\frac{3.5}{\rho_{NaCl}}+\frac{96.5}{\rho_{w}}}\\ \rho_{p}=819+1.49T-0.003T^{2} \end{array}$$

(4) $$\mu_{f,p}=5.96e^{\left(-\frac{T}{32.77}\right)}+2.3\times 10^{-4},$$

where ρ is the density (kg/m^3^), u is the velocity vector (m/s), p is the dynamic pressure (Pa), μ is the viscosity (N·s/m^2^), and *T* is the inlet temperature (K) in both channels. I, F, and ∇ represent the unit tensor, the volume force vector, and the dell operator, respectively. The density of both channels was calculated according to Equation (3), and the viscosity of both channels was calculated using Equation (4).

The heat transfer through the membrane and the boundary layers was calculated for the convection-conduction as follows [45]:

(5) $$\rho C_{p}u\cdot \nabla T+\nabla \cdot q=Q,$$

(6) $$q=-k_{m}\nabla T,$$

(7) $$\{\begin{array}{c} k_{f}=0.64\\ k_{m}=\varepsilon k_{g}+\left(1-\varepsilon \right)k_{s}\;\\ k_{p}=0.6,\;\mathrm{and} \end{array}$$

(8) $$\{\begin{array}{c} k_{g}=0.0144-2.16\times 10^{-5}T_{m}+1.32\times 10^{-7}T_{m}{}^{2}\\ k_{s}=0.178, \end{array}$$

where $C_{p}$ is the heat capacity (J/[kg·K]), q is the heat flux (W/m^2^), Q is the heat source (W/m^3^), and $k_{m}$ is the thermal conductivity of the membrane (W/[m·K]), respectively. ε is the membrane’s porosity. $k_{m}$ was calculated using Equation (7). $k_{g}$ and $k_{s}$ refer to the thermal conductivity coefficients of the vapor within the membrane’s pores and solids, respectively, as in Equation (8), and $T_{m}$ is the mean temperature.

Combined Knudsen and Poiseuille equations were used to calculate the mass transfer through the membrane’s pores, as follows [46]:

(9) $$\nabla \cdot \left(-D_{m}\nabla c\right)=0,$$

(10) $$D_{m}=\frac{\varepsilon }{\tau }\left(D_{k}+D_{p}\right),$$

(11) $$D_{k}=97\frac{d_{p}}{2}\sqrt{\frac{T}{M}}$$

(12) $$D_{p}=\frac{P\times {\left(\frac{d_{p}}{2}\right)}^{2}}{8\times \mu },$$

where c is the concentration of water (mol/m^3^), $D_{k}$ is the Knudsen diffusion coefficient (m^2^/s), $D_{p}$ is the Poiseuille flow coefficient (m^2^/s), and ε and τ are the porosity and tortuosity of the membrane, respectively. $D_{k}$ $\mathrm{and}\;D_{p}$ are calculated using Equations (11) and (12), respectively. $d_{p}$ is the membrane pore size, and *M* is the water (vapor) molecular weight (g/mol). In the Poiseuille flow equation, *P* is the mean pressure (Pa), and μ is the water (vapor) viscosity (N·s/m^2^).

The saturated pressure of the vapor was calculated using the Antoine equation, as follows [47]:

(13) $$p^{sat}=133.322*10^{\left(8.10765-\frac{1750.286}{T-38.15}\right)},$$

(14) $$\{\begin{array}{c} C_{fm}=a_{w}x_{w}\frac{p^{sat}}{RT}\\ C_{mp}=\frac{p^{sat}}{RT} \end{array}$$

(15) $$\{\begin{array}{c} a_{w}=1-0.5x_{NaCl}-10x_{NaCl}{}^{2}\\ x_{w}=1-x_{NaCl}, \end{array}$$

where $C_{fm}$ and $C_{mp}$ are the feed–membrane and membrane–permeate concentrations, respectively. $a_{w}$ is the water activity in a NaCl solution, $x_{w}$ is the liquid molar fraction of water, $x_{NaCl}$ is the liquid molar fraction of a NaCl solution, and R is the gas constant (J/[mol·K]).

### 2.2. Temperature Polarization Coefficient

The TPC can be used to quantify the magnitude of the boundary layer resistances relative to the total heat transfer resistance. In MD, the TPC is related to the thermal efficiency of the process. Therefore, this study used the CFD model to calculate the TPC, as follows [48,49]:

(16) $$\mathrm{TPC}=\frac{T_{fm}-T_{pm}}{T_{fb}-T_{pb}},$$

where $T_{fm}$ and $T_{pm}$ represent the feed and permeate in the fluid’s temperatures on the membrane’s surface, and $T_{fb}$ and $T_{pb}$ represent the bulk in the fluid temperatures of the feed and permeate channels, respectively.

### 2.3. Analysis of Exergy Destruction

Exergy destruction occurs because of the temperature difference between hot and cold media with temperatures *T*_1_ and *T*_2_, respectively, and the resultant irreversibility can be calculated according to [41], as follows:

(17) $$E_{D}=\left(1-\frac{T_{0}}{T_{1}}\right)Q_{T}-\left(1-\frac{T_{0}}{T_{2}}\right)Q_{T},$$

where *Q_T_* is the total heat transferred by the membrane and *T*_0_ is the temperature of the environment. Equation (17) above indicates that the exergy destruction is related to the temperature difference. On the feed side, the TP phenomenon causes the temperature on the membrane to be lower than that in the bulk phase. A further reduction in temperature occurs in the membrane because of its heat transfer resistance. On the permeate side, the temperature on the membrane is lower than that in the bulk phase owing to both TP and cooling phenomena. Therefore, the magnitudes of exergy destruction in the feed, membrane, and permeate can be assessed using Equations (18)–(20), respectively, as follows:

(18) $$E_{feed}=\left(1-\frac{T_{0}}{T_{fb}}\right)Q_{T}-\left(1-\frac{T_{0}}{T_{fm}}\right)Q_{T}:\;\mathrm{exergy}\;\mathrm{destruction}\;\mathrm{on}\;\mathrm{the}\;\mathrm{feed}\;\mathrm{side},$$

(19) $$E_{membrane}=\left(1-\frac{T_{0}}{T_{fm}}\right)Q_{T}-\left(1-\frac{T_{0}}{T_{pm}}\right)Q_{T}:e\mathrm{xergy}\;\mathrm{destruction}\;\mathrm{in}\;\mathrm{the}\;\mathrm{membrane},\;\mathrm{and}$$

(20) $$E_{permeate}=\left(1-\frac{T_{0}}{T_{pm}}\right)Q_{T}-\left(1-\frac{T_{0}}{T_{pb}}\right)Q_{T}:\;\mathrm{exergy}\;\mathrm{destruction}\;\mathrm{on}\;\mathrm{the}\;\mathrm{permeate}\;\mathrm{side}.$$

## 3. Materials and Methods

**Figure 2:**
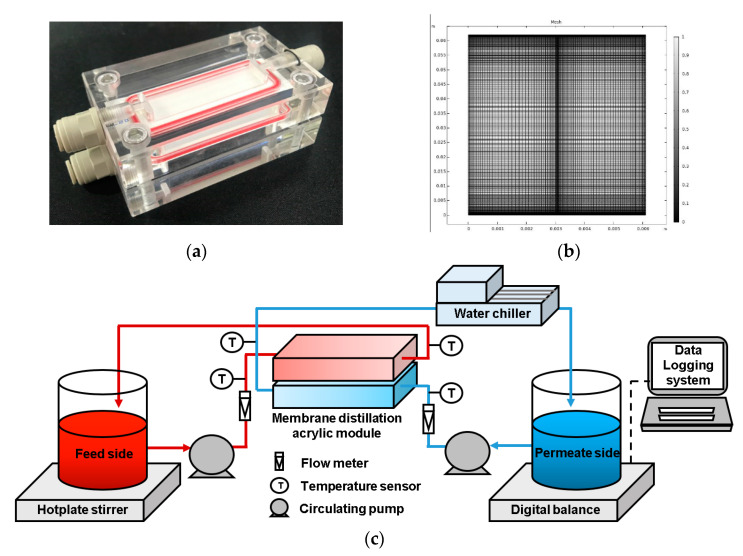
(**a**) Acrylic DCMD module, (**b**) model geometry and meshes used for the CFD simulation, and (**c**) schematic diagram of lab-scale experimental setup.

To verify the CFD model, this study conducted a set of DCMD experiments using a plate-and-frame membrane module. As the experimental details are described in the authors’ previous work [16], only a brief outline is included here. The module was designed with two channels, and the experiment was conducted using a counter-current flow. Figure 2a depicts the DCMD module, which was fabricated from acrylic to ensure chemical resistance. The channel was 60 mm in length, 15 mm in width, and 1 mm in height. Commercially available hydrophobic porous PVDF flat sheet membranes (MERCK Millipore Ltd. Burlington, MA, USA) were used. The membrane’s effective area was 900 mm^2^, its pore size was 0.22 μm, its porosity was 0.75%, and its tortuosity was 2.

Figure 2b presents a schematic diagram of the experimental setup. The feed solution and product water were circulated using a gear pump (Cole-Parmer, Chicago, IL, USA). A hotplate (IKA C-MAG, IKA, Staufen, Germany) was heated constantly to the feed-side temperature, while the product water was cooled to the temperature of the permeate side by a water chiller (JEIO TECH, Daejeon, Korea). The temperatures and flow rates of the feed and permeate were measured using flow meters and temperature sensors, respectively, and the DCMD system was operated under a counter-current mode. The volume of product water was calculated by measuring the weight of the product tank on an electronic balance (OHAUS, Lakewood, NJ, USA), and the calculations were conducted in real time based on the area of the membrane.

The CFD model was simulated using COMSOL^®^ Multiphysics 5.6 commercial software. A grid independence test was conducted to assess the optimal grid resolution. Based on this test, the number of meshes for the CFD model was determined to be 14,000. Figure 2c illustrates the model geometry and meshes used for the CFD simulation. Table 1 presents a summary of the key parameters for the model. After the model was verified, a series of CFD simulations were conducted under different operating conditions (see Table 2 for details).

## 4. Results

### 4.1. Verification of the Computational Fluid Dynamics Model

**Figure 3:**
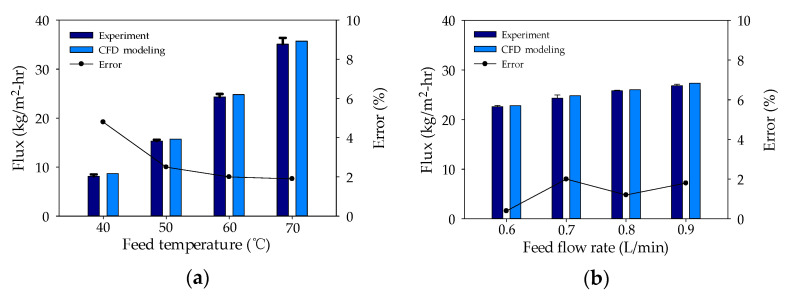
Validation of CFD model, experimental water flux results, and error rate: (**a**) difference in feed temperatures and (**b**) difference in feed flow rates.

To validate the CFD simulation, the flux calculated by the CFD model was compared with the flux measured under different feed temperature parameters (40–60 °C) and feed flow rates (0.6–0.9 L/min). It is evident in Figure 3a,b that the model calculations and the experimental data correspond well. The average error was estimated at 2.01%, indicating the suitability of the CFD model for further analysis.

### 4.2. Velocity Distribution

**Figure 4:**
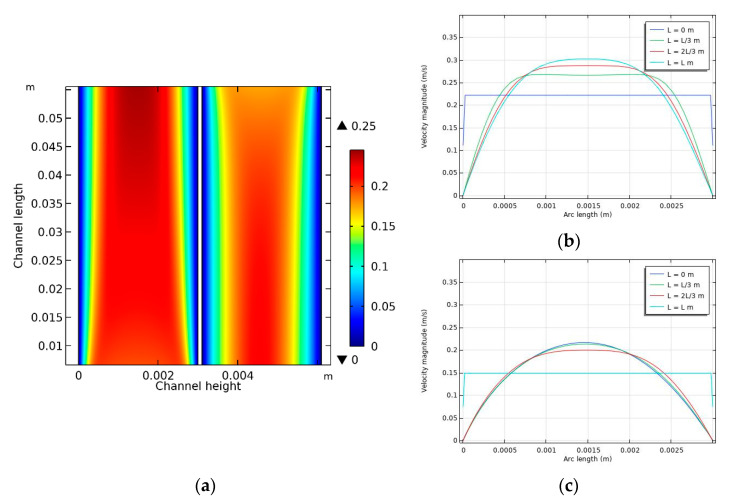
Flow velocity distribution inside the module: (**a**) velocity contour in the feed and permeate sides, (**b**) feed side, and (**c**) permeate side. Conditions: feed temperature = 60 °C, feed flow rate = 0.6 L/min, and permeate flow rate = 0.4 L/min.

The hydrodynamic conditions in the DCMD module were simulated for each case using the CFD model. An example (case 3 in Table 2) of such a simulation is presented in Figure 4. Here, the parameters for the feed temperature, feed flow rate, permeate temperature, and permeate flow rate are 60 °C, 0.6 L/min, 20 °C, and 0.4 L/min, respectively. The velocity fields in the feed and permeate channels of the DCMD system are presented in Figure 4a. The feed and permeate become fully developed as they move along the channels (*x*-axis). Accordingly, the differences in the flow velocity across the channel height (*y*-axis) increase from the inlet to the outlet. In other words, the velocity at the center of the channel is higher near the outlet than near the inlet. This is confirmed in Figure 4b,c, where the maximum velocities in the feed and permeate channels are approximately 0.3 and 0.21 m/s, respectively. In a DCMD system, this difference in velocity profiles inside the module is expected to influence the transfer of heat and mass.

### 4.3. Temperature Distribution

**Figure 5:**
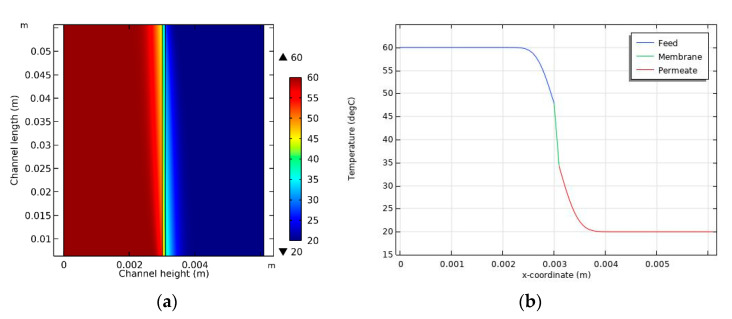
(**a**) Temperature distribution inside the DCMD module and (**b**) temperature variation according to channel height in the middle of the module (y = 0.03 m).

Because of the transfer of heat from the feed to the permeate, there may be a variation in module temperatures at different locations. The temperature distribution for case 3 is illustrated in Figure 5a. In the bulk phase, the feed temperature is close to 60 °C, but it decreases near the membrane’s surface. Conversely, the temperature of the permeate is 20 °C, which increases near the surface of the membrane. This demonstrates the TP phenomenon. In the feed, the difference between the temperature of the bulk and the membrane surface increases as the distance from the feed inlet also increases. However, in the permeate, the opposite trend is evident. Figure 5b shows the temperature profile across the height of the channel in the middle of the module (y = 0.03 m). Although there is a temperature difference of 40 °C between the feed and the permeate, there is a temperature difference of 14 °C between the feed and permeate sides of the membrane’s surface, which represents the effective temperature difference related to the net driving force. Consequently, this result indicates that the TP phenomenon significantly reduces the efficiency of the DCMD system.

### 4.4. Vapor Pressure and Flux

**Figure 6:**
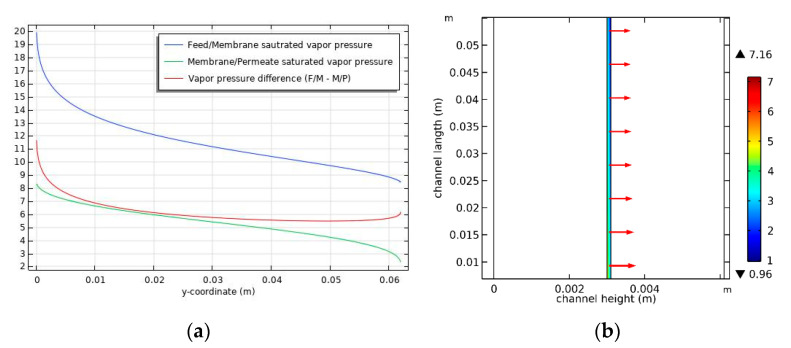
(**a**) Vapor pressure (kPa) inside the DCMD module in the membrane side, and (**b**) flux distribution. Conditions: feed temperature = 60 °C, feed flow rate = 0.6 L/min, and permeate flow rate = 0.4 L/min.

Figure 6a shows the relationship between the differences in vapor pressure according to the distance from the feed inlet. As the distance from the feed inlet increases, the net vapor pressure difference between the feed and the permeate (marked in red) decreases and then slightly increases. This is attributed to temperature changes in the feed and permeate. As shown in Figure 5a, the high feed temperature on the membrane’s surface near the inlet results in high vapor pressure. As the distance from the inlet increases, the feed temperature decreases, which reduces the vapor pressure. However, the vapor pressure increases near the feed outlet because of the relatively low permeate temperature on the surface of the membrane (Figure 5a). As shown in Figure 6b, variations in the vapor pressure difference cause the flux trend to decrease and then slightly increase with distance from the feed inlet.

### 4.5. Temperature Polairzation (TP) Phenomenon

**Figure 7:**
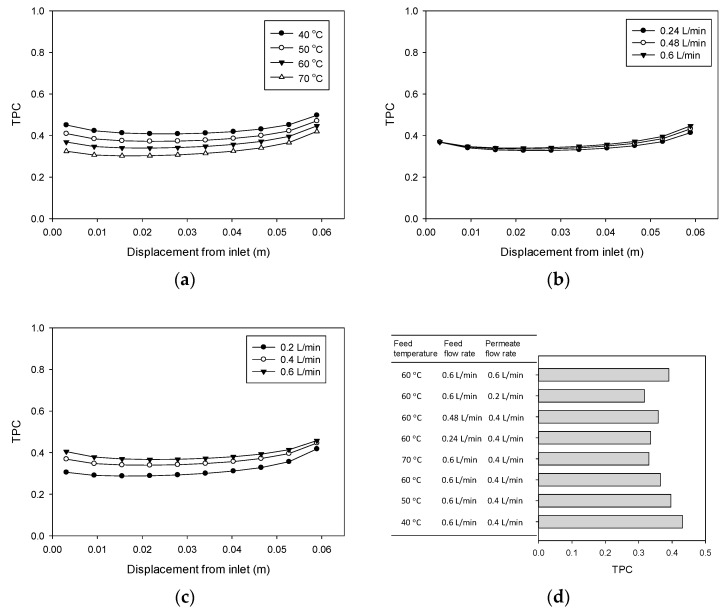
TP ratio inside the DCMD module: (**a**) effect of feed temperature, (**b**) effect of feed flow rate, (**c**) effect of permeate flow rate, and (**d**) comparison of average TP ratios under various conditions.

The TPC was calculated for each case in Table 2 based on the CFD results for temperature distribution. The TPC profiles for different feed temperature conditions in Figure 7a correspond to cases 1 to 4 in Table 2. It can be seen that in all cases, the TPC decreases from the feed inlet and then increases to the feed outlet. However, the TPC values differ according to different cases. As the feed temperature increases, the TPC tends to decrease, which indicates that TP becomes more significant. This is attributed to the fact that under high-temperature conditions, the flux is also high. Since the heat transfer rate increases with flux, the temperature difference between the bulk phase and the membrane’s surface also increases, thereby raising the TPC.

The effect of the feed flow rate on TPC profiles (corresponding to cases 3, 5, and 6) is illustrated in Figure 7b. With an increase in feed flow rate from 0.24 to 0.6 L/min, the TPC increases slightly, which results in a small reduction in TP. This can be explained by the reduction in the thickness of the boundary layer as the feed velocity increases. Consequently, the heat transfer coefficient of the boundary layer rises, leading to decreased TP. Similar trends are observed for cases 3, 7, and 8 under conditions of increased permeate flow rate. However, as Figure 7c reveals, the effect of the permeate flow rate on TPC values is greater than that of the feed flow rate. This indicates that for the DCMD module considered in this study, controlling the hydrodynamic conditions in the permeate side is more important than in the feed side. Figure 7d presents the average TPC value for each case, which is highest for case 1 (feed temperature = 40 °C) and lowest for case 7 (permeate flow velocity = 0.2 L/min).

### 4.6. Exergy Destruction Profiles

**Figure 8:**
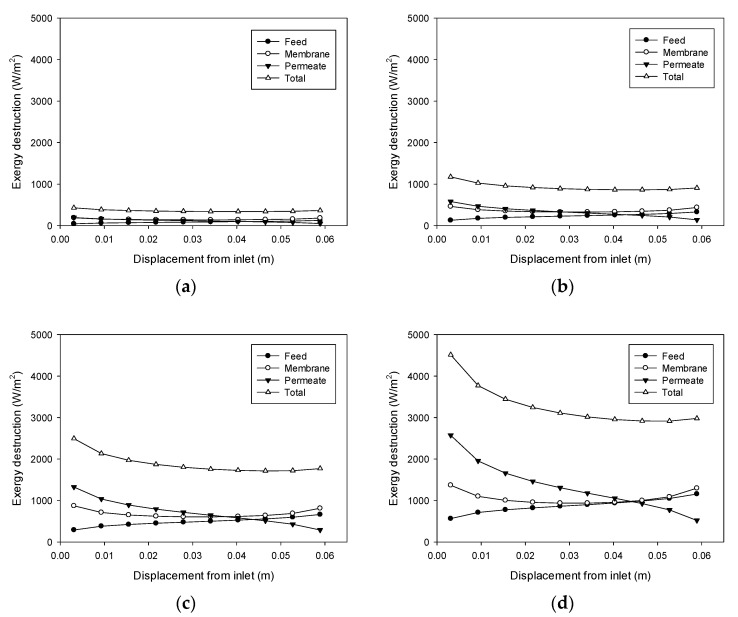
Profiles of exergy destruction in the DCMD module: (**a**) Feed temperature = 40 °C, feed flow rate = 0.6 L/min, and permeate flow rate = 0.4 L/min; (**b**) Feed temperature = 50 °C, feed flow rate = 0.6 L/min, and permeate flow rate = 0.4 L/min; (**c**) Feed temperature = 60 °C, feed flow rate = 0.6 L/min, and permeate flow rate = 0.4 L/min; (**d**) Feed temperature = 70 °C, feed flow rate = 0.6 L/min, and permeate flow rate = 0.4 L/min.

According to Equations (18)–(20), exergy destruction can be calculated using the CFD results for the heat transfer rate and the temperatures on both sides of the membrane. The exergy destruction values of the feed, membrane, and permeate were estimated individually to obtain the total exergy destruction values. Figure 8 shows the profile change for exergy destruction according to feed temperature. When the feed temperature is 40 °C (Figure 8a), the exergy destruction is relatively low. The greatest exergy destruction occurs in the membrane, which accounts for 43% of total exergy destruction. At a feed temperature of 50 °C (Figure 8b), exergy destruction is higher than the previous case at 40 °C, which is attributed to an increase in the heat transfer rate commensurate with flux. The heat transfer across the membrane results from the latent heat of the water vapor passing through the pores. With a higher feed temperature, the flux increases to enhance the heat transfer process, thereby increasing the destruction of exergy. As the feed temperature increases to 50 °C (Figure 8c) and 60 °C (Figure 8d), additional exergy destruction occurs.

Figure 8d reveals that the destruction of exergy in the feed rises as the distance from the feed inlet increases. Conversely, there is a decrease in exergy destruction in the permeate as the distance from the feed inlet increases. The exergy destruction in the membrane exhibits a parabolic profile. Consequently, the total destruction of exergy decreases and becomes almost constant. These findings suggest that the main reason for the destruction of exergy differs according to the location in the module. The TP phenomenon occurring in the feed could be an important influence on the exergy destruction near the feed outlet, while that occurring in the permeate may be an important effect closer to the feed inlet.

**Figure 9:**
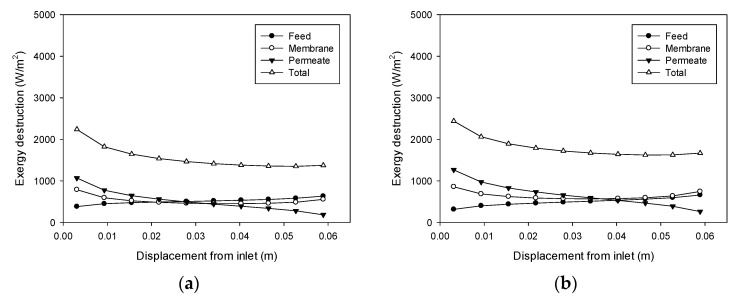
Profiles of exergy destruction in the DCMD module: (**a**) Feed temperature = 60 °C, feed flow rate = 0.24 L/min, and permeate flow rate = 0.4 L/min; (**b**) Feed temperature = 60 °C, feed flow rate = 0.48 L/min, and permeate flow rate = 0.4 L/min.

Figure 9 illustrates the effect of the feed flow rate on exergy destruction (cases 5 and 6). According to Figure 7b, the feed flow rate does not have a significant effect on the TPC. Therefore, its effect on exergy destruction is also insignificant, and only a small increase is observed as the feed flow rate rises.

**Figure 10:**
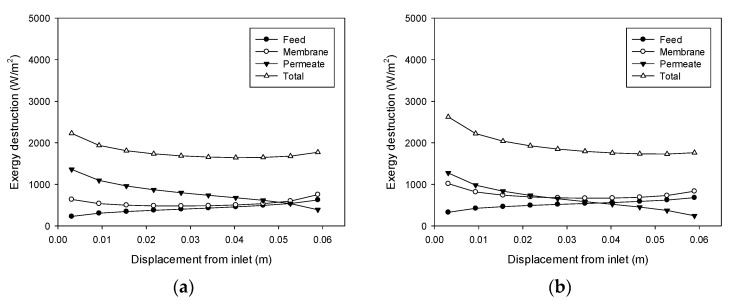
Profiles of exergy destruction in the DCMD module: (**a**) Feed temperature = 60 °C, feed flow rate = 0.6 L/min, and permeate flow rate = 0.2 L/min; (**b**) Feed temperature = 60 °C, feed flow rate = 0.6 L/min, and permeate flow rate = 0.6 L/min.

Figure 10 shows the influence of the permeate flow rate on exergy destruction, which corresponds to case 7 and 8. Exergy destruction increases with an increase in the permeate flow rate. The effect of the permeate flow rate on TPC is greater than that of the feed flow rate, which is shown in Figure 7. Accordingly, an increase in the permeate flow rate results in a larger increase in exergy destruction compared with the case of the feed flow rate.

### 4.7. Exergy Flow Analysis

**Figure 11:**
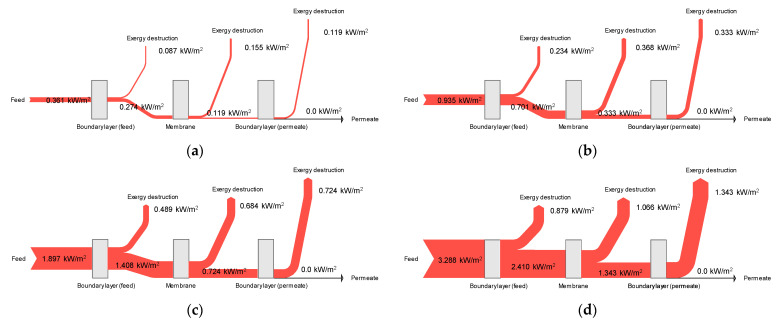
Exergy flow analysis for the DCMD module: (**a**) Feed temperature = 40 °C, feed flow rate = 0.6 L/min, and permeate flow rate = 0.4 L/min; (**b**) Feed temperature = 50 °C, feed flow rate = 0.6 L/min, and permeate flow rate = 0.4 L/min; (**c**) Feed temperature = 60 °C, feed flow rate = 0.6 L/min, and permeate flow rate = 0.4 L/min; (**d**) Feed temperature = 70 °C, feed flow rate = 0.6 L/min, and permeate flow rate = 0.4 L/min.

To assess the energy efficiency of DCMD systems, it is essential to understand the flow of exergy inside such schemes. Accordingly, this study analyzed exergy flows based on the results of CFD modeling. The average exergy destructions in the feed, membrane, and permeate were calculated to establish an exergy balance. Figure 11 shows the exergy flows at different feed temperature conditions for cases 1–4. Since the permeate temperature and the atmospheric temperature are identical, no exergy remains in the permeate. This suggests that the total exergy in the feed is destructed in the feed boundary layer, the membrane, and the permeate boundary layer; e.g., the total exergy is 0.935 kW/m^2^ in Figure 11a. The feed, membrane, and permeate account for 24.1%, 43.0%, and 32.9% of exergy destruction levels, respectively. Conversely, the total exergy in Figure 11d is 3.288 kW/m^2^. The exergy destruction contributions from the feed, membrane, and permeate are 26.7%, 32.4%, and 40.8%, respectively. These results indicate that the relative importance of the exergy destruction mechanism is affected by different operating conditions. It is expected that the CFD-based approach for calculating exergy destruction will be a useful tool for analyzing and optimizing MD systems.

## 5. Conclusions

This study developed a CFD model to analyze the hydrodynamic, heat transfer, and mass transfer phenomena in DCMD systems. Based on the CFD results, the TPC and exergy destruction magnitude were estimated by varying the feed temperature from 40 °C to 70 °C. Moreover, the effect of the flow rate on the TPC and exergy destruction was examined by adjusting the flow rate from 0.24 L/min to 0.6 L/min. Within the module, the throughflow of feed and permeate causes the thickening of the boundary layer, which affects the TP. In this study, the TPC ranged from 0.3178 to 0.4312 under the considered conditions. The TP was found to be the least severe under a low feed temperature (case 1), while it was the most significant under conditions of low permeate velocity (case 7). Furthermore, the operating conditions were confirmed to affect exergy destruction patterns. Exergy destruction mechanisms within the permeate were found to be the most significant close to the feed inlet, although they became insignificant nearer the feed outlet. The opposite trend was observed for exergy destruction in the feed. Therefore, this study confirmed that the relative importance of exergy destruction mechanisms is affected by operating conditions. Although the present work elucidated exergy flows in a DCMD module based on CFD simulations, it can be extended to other MD modules such as AGMD, SGMD, and VMD. Moreover, it is recommended to apply 3D CFD models to further improve the accuracy of the exergy calculations. These will bring new opportunities for thermodynamic optimization of various MD modules and processes.

## Figures and Tables

**Table 1:** Summary of CFD model parameters.

Parameter	Value
Feed channel height	0.003 m
Permeate channel height	0.003 m
Channel length	0.06 m
Heat of evaporation	2.333 × 10^6^ [W·s]/kg
Feed thermal conductivity	0.64 W/[m·K]
Permeate thermal conductivity	0.6 W/[m·K]
Membrane thermal conductivity	0.04 W/[m·K]
Membrane pore size	0.22 μm
Membrane thickness	100 μm
Membrane tortuosity	2

**Table 2:** Operating conditions for CFD simulation cases.

Case	Feed Side		Permeate Side	
	Temperature (°C)	Flow Rate (L/min)	Temperature (°C)	Flow Rate (L/min)
1	40	0.6	20	0.4
2	50			
3	60			
4	70			
5	60	0.24		
6		0.48		
7		0.6		0.2
8		0.6		0.6

## Data Availability

Not applicable.

## Nomenclature

$a_{w}$	Water activity in NaCl solution
c	Concentration of water, mol/m^3^
$C_{fm}$	Feed-membrane concentration, mol/m^3^
$C_{mp}$	Membrane-permeate concentration, mol/m^3^
$C_{p}$	Heat capacity, J/[kg·K]
$d_{p}$	Membrane pore size, m
$D_{k}$	Knudsen diffusion coefficient, m^2^/s
$D_{p}$	Poiseuille flow coefficient, m^2^/s
*E*_*D*_	Energy destruction, kW/m^2^
*E*_*feed*_	Exergy destruction on the feed side, kW/m^2^
*E*_*membrane*_	Exergy destruction in the membrane, kW/m^2^
*E*_*permeate*_	Exergy destruction on the permeate side, kW/m^2^
F	Volume force vector
I	Unit tensor
$k_{m}$	Thermal conductivity coefficients of membrane, W/[m·K]
$k_{g}$	Thermal conductivity coefficients of vapor, W/[m·K]
$k_{s}$	Thermal conductivity coefficients of solid, W/[m·K]
M	Water(vapor) molecular weight, g/mol
p	Dynamic pressure, Pa
P	Mean pressure, Pa
q	Heat flux, W/m^2^
*Q*	Heat source, W/m^3^
*Q*_*T*_	Total heat transferred by the membrane, kW
*R*	Gas constant, J/[mol·K]
*T*	Temperature, K
*T*_0_	Temperature of the environment, °C
$T_{m}$	Mean temperature, K
$T_{fm}$	Feed temperature on the membrane surface, °C
$T_{pm}$	Permeate temperature on the membrane surface, °C
$T_{fb}$	Feed temperature in bulk fluid, °C
$T_{pb}$	Permeated temperature in bulk fluid, °C
u	Velocity vector, m/s
$x_{w}$	Liquid mole fraction of water
$x_{NaCl}$	Liquid mole fraction of NaCl solution
ρ	Density, kg/m^3^
μ	Viscosity, N·s/m^2^
ε	Porosity of the membrane
τ	Tortuosity of the membrane
∇	Dell operator
